# Microbiota and mucosal gene expression of fecal microbiota transplantation or placebo treated patients with chronic pouchitis

**DOI:** 10.1080/19490976.2023.2295445

**Published:** 2024-01-12

**Authors:** Anna K. Hartikainen, Imran Khan, Essi K. Karjalainen, Laura Renkonen-Sinisalo, Perttu Arkkila, Jonna Jalanka, Anna H. Lepistö, Reetta Satokari

**Affiliations:** a Human Microbiome Research Program, Faculty of Medicine, University of Helsinki, Helsinki, Finland; b Department of Gastrointestinal Surgery, Helsinki University Hospital, Helsinki, Finland; c Genome-Scale Biology Research Program, Faculty of Medicine, University of Helsinki, Helsinki, Finland; d Department of Gastroenterology, Helsinki University Hospital, Helsinki, Finland; e Department of Medicine, University of Helsinki, Helsinki, Finland

**Keywords:** Fecal microbiota transplantation, ulcerative colitis, pouchitis, inflammatory bowel disease, microbiota, gene expression

## Abstract

Altered microbiota and impaired host immune function have been linked to the pathogenesis of pouchitis. We used 16S rRNA gene sequencing and RNA sequencing data from a previous randomized clinical trial (RCT) on fecal microbiota transplantation (FMT) therapy in 26 chronic pouchitis patients with one-year follow-up. We analyzed changes in both luminal and mucosal microbiota composition, as well as in host mucosal gene expression to gain insights into the host–microbiota interactions possibly underlying clinical outcomes of the patients. Antibiotic type and pattern of use were significant drivers of the luminal microbiota at baseline. Differential gene expression analysis indicated transition from ileal to colonic gene expression in the pouch, and upregulation in inflammation- and immune system-related pathways in the pouch. At 4 weeks, the non-relapsed FMT patients had a lower microbiota dissimilarity to the donor than the non-relapsed placebo patients (*p* = .02). While two FMT-treated patients showed a shift toward the donor’s microbiota during the one-year follow-up, the overall FMT microbiota modulation effect was low. Patient’s luminal and mucosal microbiota profiles were unstable in both FMT and placebo groups. Expression of the chemokine receptor CXCR4 was downregulated at 52 weeks compared to the baseline in the non-relapsed patients in both FMT and placebo groups. Microbiota modulation by FMT seems to be low in this patient group. The microbiota composition or alterations did not explain the relapse status of the patients. Some evidence for remission-related host gene expression pattern was found; specifically, CXCR4 expression may have a role in sustained remission.

## Introduction

Human gut microbiota has a critical role in the maintenance of immunological homeostasis in the intestinal tract. Accordingly, impairment of these sensitive host–microbe interactions has been linked to altered immune responses and autoimmune diseases.^1,2^ For example, inflammatory bowel disease (IBD) is a chronic debilitating disorder of multifactorial etiology, consisting of two main conditions – ulcerative colitis (UC) and Crohn’s disease (CD). Host–microbiota interaction has been shown to be disturbed in IBD, and alterations to microbiota are considered to contribute to the development and maintenance of inflammation in IBD.^3^ An ileal pouch-anal anastomosis (IPAA) is the gold standard surgical intervention for severe UC,^4^ when, for example, treatment with conventional therapy using anti-inflammatory medication, immunosuppressants, or biologics has failed. However, approximately half of the UC patients who undergo IPAA develop pouchitis in the first 10 years after surgery, and approximately 15–20% of the patients suffer from chronic pouchitis.^5–7^ The disease etiology is still partly unknown, and treatment of chronic pouchitis is challenging.^8,9^

There is evidence that microbiota plays a pivotal role in the pathogenesis of pouchitis. Previous research suggests that pouchitis patients have reduced luminal and mucosal bacterial diversity and richness as compared to healthy controls.^10,11^ However, much less is known about specific bacterial profiles that would characterize the microbiota composition in pouchitis.^12^ In addition to microbiota, host genetics seem to have an impact on the risk of the disease, as pouchitis is more common in patients with UC than familial adenomatous polyposis (FAP).^13^ Changes in the mucosal gene expression are reported early after the IPAA surgery, and a higher correlation with “colon-like” gene expression in the ileal pouch mucosa may be connected to a higher risk for pouchitis.^14^ Alterations in the pouch microbiota, together with genetic susceptibility and reprogramming of gene expression in the mucosa, seem to underlie the disease persistence.

Treatments that can modulate patients’ microbiota have been proposed regarding microbiota composition differences among those suffering from pouchitis.^15^ Antibiotics such as ciprofloxacin or metronidazole are used to treat both acute and chronic pouchitis. Moreover, a probiotic product (VSL#3) has been shown to help maintain remission.^16^ Fecal microbiota transplantation (FMT) is an effective treatment for recurrent *Clostridioides difficile* infection (rCDI).^17,18^ Here, the introduction of a healthy microbiota into the intestinal tract of rCDI patients offers colonization resistance against *C. difficile*.^19^ Similarly, it has been suggested that FMT might be effective in correcting the dysbiosis in other indications such as pouchitis, and lead to a clinical success. Results on the treatment of IBD by FMT have been promising yet inconsistent across different clinical trials, possibly due to variation in the FMT protocols.^20^ Concerning pouchitis, previous studies on FMT are limited mostly to case studies, small cohorts, or have only a short follow-up time. Moreover, the knowledge on microbiota changes after FMT in pouchitis is scarce.

Recently, we conducted the first completed randomized placebo-controlled trial on the treatment of chronic pouchitis by FMT.^21^ Although the overall clinical outcome of the trial was negative, the frequent sampling conducted during a one-year follow-up period from this trial provided us with unique material to study the characteristics of pouchitis microbiota in the long term. The aim of this study was to investigate the luminal and mucosal microbiota of chronic pouchitis patients. The patients were followed for one year after FMT or placebo treatment, with the aim of evaluating the success of donor microbiota engraftment and associating differences in the microbiota with the clinical outcomes. Moreover, we investigated the host mucosal gene expression in the ileum and the pouch, both at baseline and one year after the FMT treatment. We hypothesized that FMT-treated patients with a positive outcome would have a successful bacterial engraftment from the donor, while relapsing patients would show distinctive bacterial signatures preceding the relapse. In addition, we hypothesized that host mucosal gene expression would be affected by the FMT treatment, and that the transcriptomic profile of relapsing patients would differ from that of non-relapsing patients.

## Materials and methods

### Clinical trial and donor selection

The clinical trial has previously been described in more detail.^21^ Briefly, 26 chronic pouchitis patients were randomized in a 1:1 ration into FMT and placebo groups. The patients received two FMTs (the first via pouchoscopy, and the second one was administered through a catheter four weeks later) either from a healthy donor or autologous transplants, which served as the placebo. We used a single donor approach; the donor was a 52-year-old female who had not received antibiotics or probiotics six months prior to the donations. The donor was screened according to the international guidelines for donor screening for pathogens, to exclude the potential risks related to FMT treatment.^18,22^ The fecal suspensions from the donor were prepared and stored frozen as previously described.^23^ Altogether six stool samples were collected from the donor over an eight-month period for the microbiota analysis. Patients provided autologous transplants at the day of the procedure, and these were given as fresh transplants.

We collected stool samples from patients at baseline, and again at 4, 12, 26, and 52 weeks. All samples were stored at −80°C until processing. Altogether, 12 stool samples were not provided. Mucosal biopsy samples were taken with biopsy forceps from the ileum and pouch at baseline and at 52 weeks. Biopsies were stored in RNAlater (Invitrogen by Thermo Fisher Scientific, CAT#AM7020) at 4°C for the first 24 hours, and then at −20°C until processing. In total, all 104 mucosal samples were collected.

Patients were advised to discontinue all antibiotic treatments 36 hours before the first transplantation. However, they were allowed to continue the use of probiotic products during the trial. Four patients in the placebo group and five patients in the FMT group used probiotic products at some point during the follow-up period. Three out of five patients in the FMT group used probiotic VSL#3 (Vivomixx) continuously throughout the trial. Relapse was defined as a need for antibiotics and the return of the usual pouchitis symptoms. If relapse occurred before the second administration of FMT, patients were advised to discontinue the use of antibiotics 36 hours before the second procedure.

Pouchitis Disease Activity Index (PDAI) was measured at baseline and at 52 weeks. In addition, the clinical PDAI (cPDAI) without histological and endoscopy results, as well as fecal calprotectin were measured at all five time points listed above. All patients gave written informed consent, and the clinical trial was approved by the institutional review board and ethics committee of the Helsinki University Hospital (HUS/2789/2017). The trial was registered at ClinicalTrials.gov with the identifier NCT03378921.

### DNA extraction and sequencing

All fecal samples were stored at −80°C until DNA extraction. We extracted DNA with a validated method including repeated bead beating as a mechanical lysis step.^24,25^ KingFisher Flex 96 (Thermo Fisher Scientific, CAT#5400610) was used for high-throughput DNA extraction, and DNA concentrations were measured with a Quant-iT™ dsDNA Assay High Sensitivity (HS) kit (Invitrogen, Thermo Fisher Scientific, CAT#Q33120) following the manufacturer’s protocol.

Microbiota composition in stool samples was analyzed by 16S rRNA gene sequencing (MiSeq, Illumina) with primers targeting the V3-V4 hypervariable region of the 16S rRNA gene. The library preparation for fecal DNA samples was conducted with a two-step PCR (see details in Additional File 2). Next, a 20 nM pool was aliquoted and purified with AGENCOURT® AMPURE® XP beads (Beckman Coulter Life Sciences, CAT#A63881), and the concentration was measured with a Qubit™ dsDNA HS Kit (Thermo Fisher Scientific, CAT#Q32851). The pool was delivered to the sequencing laboratory (Biomedicum Functional Genomics Unit at the Helsinki Institute of Life Science and Biocenter Finland at the University of Helsinki, Finland). The sequencing data is stored at European Nucleotide Archive (ENA) with the accession number PRJEB52304.

Both ileum and pouch biopsy samples were extracted with the same previously published protocol, which includes both mechanical and chemical lysis of the cells.^26^ First, the bacterial DNA in the samples was amplified (see details in Additional File 2) and PCR products purified by exo SAP-IT Express (Thermo Fisher Scientific, CAT#75001.1.ML). The purified products were delivered for library preparation and Illumina MiSeq sequencing with forward primer (S-D-Bact-0341-b-S-17) and reverse primer (S-D-Bact-0785-a-A-21).^27^ Sequencing was conducted in the Institute for Molecular Medicine Finland, HiLIFE, Helsinki Finland. The sequencing data is stored at ENA with the accession number PRJEB52304.

### Processing of 16S rRNA gene sequence data and statistical testing

Microbiota composition was analyzed using the mare package in RStudio and R version 4.1.2 (see details in Additional File 2).^28,29^ The function “ProcessReads” was used for the forward reads that were trimmed 20 bp after the primer sequence to a length of 180 bp. Reads were also quality and chimera filtered and truncated. Reads under 0.001% relative abundance were discarded. After pre-processing, the samples with under 10 000 reads were excluded, resulting in 106/118 fecal and 102/104 mucosal samples being included in the analysis. The average read count of the sequenced fecal samples after the filtering step was 44 687 (±22 863), median 42 053. For the biopsy samples, the mean read count was 81 848 (±66 162), median 69 992. Taxonomy was assigned using the function ‘TaxonomicTable’, which used USEARCH against the Ribosomal Database Project (RDP) database version 16.^30,31^ Diversity was calculated with the R package vegan using the Inverse Simpson index.^32^

Statistical testing for microbiota data was done with the mare package in RStudio and R version 4.1.2.^28,29^ The mare package combines functions from other R packages, such as vegan, MASS, and nlme.^32–34^ Permutational analysis of variance (PERMANOVA) using genus-level Bray-Curtis dissimilarity was used to determine the possible effect of different factors on the microbiota composition. “Betadisper” function in R package vegan was used to check the homogeneity condition.^32^ Principal Coordinates Analysis (PCoA) was used to analyze beta diversity at the genus level relative abundance with the Bray-Curtis dissimilarity index. The function “GroupTest” was used for differential abundance testing between selected groups. The function fits the data for the most suitable statistical model. The model options include negative binomial models, linear models, generalized least squares models or linear mixed models. Moreover, the function uses the read count as an offset, and it corrects the initial p-values with false discovery rate (FDR) correction to q-values. Similarity to donor was measured by the Bray-Curtis dissimilarity index using genus-level data. An average Bray-Curtis dissimilarity index value of the six longitudinal donor samples was used. To evaluate the bacterial engraftment from the donor to the patients, we first determined the amount of shared operational taxonomic units (OTU) between the donor samples and patients’ pre-FMT sample. Those that were only present in the donor were classified as an OTU that could possibly be transferred to the patient. Shared OTUs were then calculated at the follow-up time points; those that were absent at patient’s baseline sample, but present in the post-FMT and donor sample were classified as potentially donor derived. ANOVA or Student’s T-test were used for data that was normally distributed, while non-parametric Wilcoxon signed rank test or Kruskal-Wallis were used for non-normally distributed data. Statistical difference was considered significant with p-value <0.05 and q-value <0.05. Figures were produced with R package ggplot2.^35^

### RNA extraction protocol

RNA was extracted from human ileum and pouch biopsy samples stored in RNAlater at −20°C (Invitrogen by Thermo Fisher Scientific, CAT#AM7020). Total RNA was extracted using the RNeasy Mini Kit (Qiagen, CAT#74104) according to the manufacturer’s instructions, and samples were homogenized using ceramic beads (Precellys). RNA quantification was done using a NanoDrop1000 Spectrophotometer (Thermo Fisher Scientific) or Qubit RNA BR assay kit (Thermo Fisher Scientific, CAT#Q10210). The RNA integrity number (RIN) values were checked using TapeStation.

### RNA sequencing protocol

We used the 3’UTR RNAseq “Bulkseq” method as described previously.^36^ This method is based on the Dropseq method for single-cell sequencing.^37^ Here, mRNA was primed with an oligo-dT primer, which also contains a 12 bp “Cell barcode” to identify the sample and an 8 bp unique molecular identifier (UMI) sequence which can be used to remove PCR duplicates during the data analysis step. After reverse transcription, single-stranded cDNA was converted into ds cDNA using the template switch effect, and the product was then amplified with a PCR (12 cycles) using SMART PCR primers. Samples were then pooled together, and PCR sequencing pools were made with Nextera i7 primers and the Dropseq P5 primer. The sequencing was performed on two NextSeq High Output 75 cycle flow cells on the Illumina NextSeq 500 platform. The sequencing was performed at the Biomedicum Functional Genomics Unit at the Helsinki Institute of Life Science and Biocenter Finland at the University of Helsinki, Finland. The RNA sequencing data (gene count table) is available by request.

### Alignment of RNA sequence reads and generation of digital expression data

First, we removed short sequence reads (<20 bp) from the raw sequence data. The data was further processed using drop-seq tools following the analysis steps as previously described in detail.^36,37^ Briefly, the reads were additionally filtered to remove polyA tails that were 6 bp or longer, then aligned to the human genome using STAR aligner with the default settings. The alignments and annotations were done using the human reference genome from GENCODE Human release version 35 (GRCh38.p13). Uniquely mapped reads were grouped according to the sample-specific barcodes, and gene transcripts were counted by their UMIs to reduce bias emerging from the PCR amplification. Digital expression matrices that report the number of transcripts per gene in each sample were also compiled with drop-seq tools. The edgeR package was used for normalization and differential expression analysis.^38^ The differentially expressed (DE) genes were selected at FDR of <0.05.

### Ingenuity pathway analysis

The significantly up- and/or downregulated genes were separately submitted to QIAGEN Ingenuity Pathway Analysis (QIAGEN IPA) software to identify significant canonical pathways.^39^ The statistical significance of the overlap of the molecules in our dataset was determined by a right-tailed Fisher’s Exact Test followed by multiple hypothesis correction with the Benjamini-Hochberg method.^40^

## Results

### Characteristics of the chronic pouchitis microbiota at baseline differed from the healthy donor

**Figure 1.**
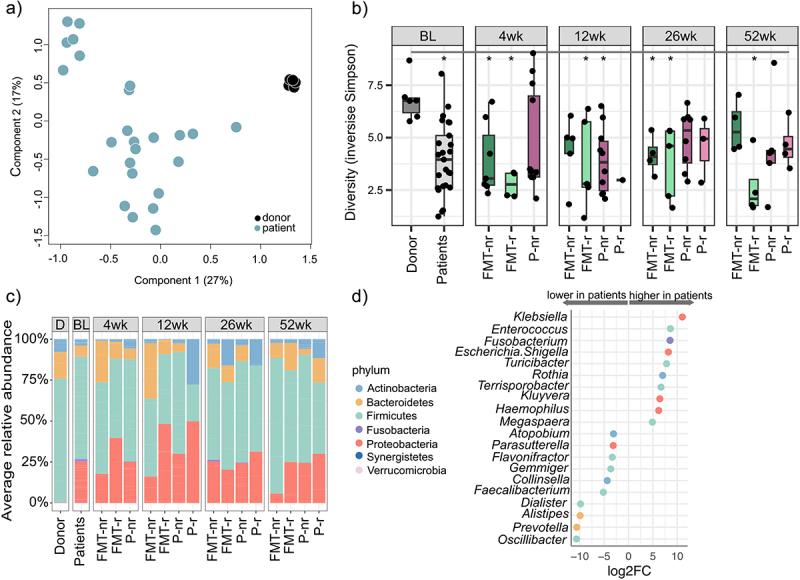
Luminal microbiota characteristics in chronic pouchitis patients, and comparison to the healthy fecal donor.

We first analyzed the luminal microbiota composition of the chronic pouchitis patients at baseline and compared it to that of the healthy donor. The microbiota of the patients and healthy donor were significantly separated in the PCoA (Figure 1a). The donor had a significantly higher bacterial diversity than the patients (Figure 1b). The phylum Proteobacteria was significantly more abundant in the pouchitis patients than in the donor (25.51% ±24.38 and 0.2% ±0.13, respectively, q < 0.05), and the abundance of Bacteroidetes was lower in the pouchitis patients as compared to the donor (6.82% ±17.65 versus 16.44% ±13.80, respectively, q < 0.05) (Figure 1c, Supplementary table S1, Additional File 1). While the average abundance of the phylum Fusobacteria was low on average in the patients (1.24% ±6.14), one patient had a surprisingly high relative abundance (30.70%). The abundance of Fusobacteria was low in the donor (0.002% ±0.004).

a: Genus-level Principal Coordinates Analysis (PCoA) plot at baseline showing separation of patients and the donor. b: Diversity index (inverse Simpson) in the donor samples and patients at baseline and follow-up time points. nr = non-relapsed, r = relapsed. Statistically significant difference indicated with an asterisk (*p* < .05). c: Phylum-level mean relative abundance in the donor and patients at baseline (BL) and follow-up time points (4 wk, 12 wk, 26 wk and 52 wk) based on study group and relapse status. nr = non-relapsed, *r* = relapsed. d: Genera with a statistically significant (q < 0.05) difference in relative abundance at baseline between pouchitis patients and the healthy donor. Selected 20 genera with the highest and lowest log2 fold change. Color of the points indicates the phylum.

At the genus level, 38 genera showed a statistically significant difference between pouchitis patients and the donor (Supplementary table S1, Additional File 1). The patients had a higher abundance of several genera in the phylum Proteobacteria, such as *Kluyvera*, *Haemophilus*, and *Escherichia/Shigella*. Moreover, genera *Clostridium IV*, *Faecalibacterium*, and *Oscillibacter* in the family *Ruminococcaceae* and genus *Prevotella* belonging to the phylum Bacteroidetes had a lower abundance in the patients than in the healthy donor (Figure 1d).

Together the results showed that pouchitis luminal microbiota was less diverse and compositionally different from healthy fecal microbiota. It was enriched in facultative anaerobes belonging to the phylum Proteobacteria, and the level of obligate anaerobes was reduced.

### Type and pattern of antibiotic use is reflected in microbiota composition

**Figure 2.**
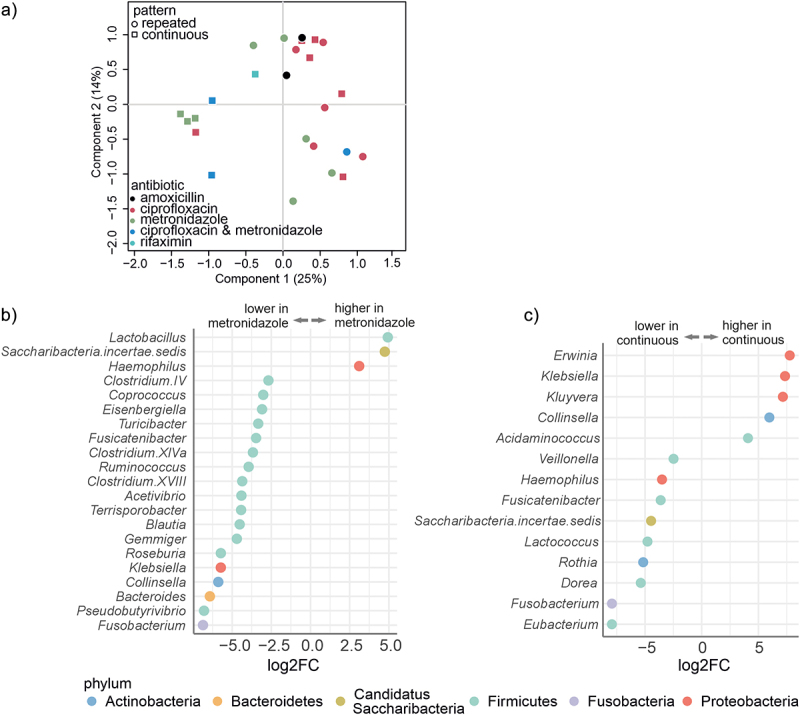
Chronic pouchitis patients had significant differences in the luminal microbiota composition at baseline, based on the pattern of the antibiotic use and type of antibiotics used.

We aimed to identify factors that may have contributed to patients’ microbiota composition at baseline before the intervention. We tested the effect of several variables on the luminal microbiota composition to identify potential predictive profiles from the microbiota. These groupings included: difference between the intervention groups, pattern of antibiotic and probiotic use, type of antibiotic, PDAI value at baseline, calprotectin, patients age, and sex, as well as age at which the patient underwent IPAA surgery, and clinical outcome (relapse or non-relapse) on the luminal microbiota at baseline. Of these, the type of antibiotic and pattern of use had a significant effect on the microbiota composition (Figure 2a). The type of antibiotic explained 23% (PERMANOVA, *p* =.032, Supplementary table S2, Additional File 1), and the pattern of antibiotic use explained 10% (PERMANOVA, *p* =.011, Supplementary table S2, Additional File 1) of the luminal microbiota variation. Of note, also the group dispersions differed significantly in the type of antibiotic (*p* =.02, betadisper, Supplementary table S2, Additional File 1). On the contrary, when we analyzed the mucosal microbiota at baseline, it was not possible to separate patients based on type or pattern of the usage of antibiotics. Also, the other tested patient variables did not explain mucosal microbiota variation in the pouch or the ileum (Supplementary table S2, Additional File 1).

a:Principal Coordinates Analysis (PCoA) plot at baseline. Patients are grouped by repeated or continuous antibiotic usage and by the type of antibiotic. b: Genera with a significant (q < 0.05) difference in the relative abundance at baseline between patients using metronidazole or ciprofloxacin. Different colors of the points correspond to different phyla. c: Genera with a significant (q < 0.05) difference in the relative abundance at baseline between patients using antibiotics repeatedly or continuously. Different color of the points corresponds to different phyla.

In the luminal microbiota, there was a significant reduction of several genera from the phylum Firmicutes and genus *Bacteroides* from the phylum Bacteroidetes among the patients who used metronidazole compared to the patients who used ciprofloxacin (Figure 2b, Supplementary Table S3, Additional File 1). Furthermore, the average relative abundance of the phylum Firmicutes was lower in the patients using continuous antibiotics than in those using repeated courses (47.53% ±26.86 and 76.37% ±21.60, respectively, q < 0.05) (Supplementary Table S4, Additional File 1). At the genus level, a total of 14 taxa were found to be statistically different in abundance between the patients using continuous or repeated antibiotics (Figure 2c). Three genera (*Erwinia*, *Klebsiella*, and *Kluyvera*) belonging to the class Gammaproteobacteria were more abundant in patients who used continuous antibiotics as compared to repetitive antibiotics users (Figure 2c).

Taken together, the baseline differences in patients’ luminal microbiota were mainly driven by the type and pattern of antibiotics use. In particular, the relative abundance of strictly anaerobic genera was decreased in the patients who used metronidazole, and some genera belonging to Gammaproteobacteria were more abundant in the patients using continuous antibiotics. However, a similar effect of the type or pattern of antibiotic use was not found in the mucosal microbiota at baseline.

### Unstable microbiota profiles and low-level similarity to donor’s microbiota during one-year follow-up

The study protocol consisted of two fecal transplantations – the first at baseline in pouchoscopy and another four weeks later via catheter – and a one-year follow-up period. We aimed to study the longitudinal patterns of pouch microbiota, as well as the transfer and persistence of the donor’s microbiota in the patients. Overall, the patients had a heterogenic and unstable luminal microbiota composition post-FMT (Figure 1c, Supplementary Figure S1, Additional File 3). However, there were no consistent bacterial profiles associated with relapse episodes. When studying the persistence of the donor’s microbiota in the patients, samples taken after relapse were excluded from the analysis, as these patients had started using antibiotics.

**Figure 3.**
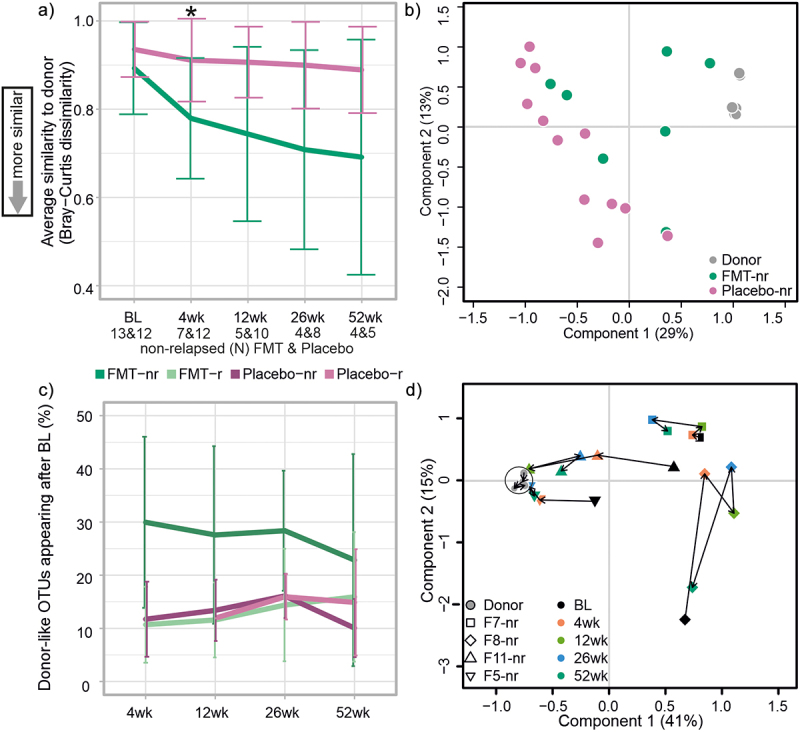
The effect of FMT on microbiota composition.

On average, dissimilarity between the FMT-treated patients and the donor decreased post-FMT, suggesting that FMT-treated patients had more similar microbiota with the donor after FMT treatment than what was determined at baseline (Figure 3a). The average dissimilarity between patients in the placebo group and the donor remained high throughout the follow-up. The microbiota dissimilarity between patients and the donor was significantly lower in the FMT group than in the placebo at the 4-week time point (Figure 3a, *p* = .02, Wilcoxon signed rank test). A similar trend continued at weeks 12 to 52, but the differences were not significant. Moreover, the luminal PCoA with Bray-Curtis dissimilarity at 4 weeks revealed that microbiota of patients in the placebo group remained distinct from that of the donor’s, while microbiota of some FMT-treated patients grouped with the donor (Figure 3b).

a: Average Bray-Curtis dissimilarity index between the non-relapsed FMT-treated patients and placebo groups compared to the donor. Non-relapsed FMT-treated patients had lower microbiota dissimilarity with the donor at week four (4 wk) than patients in the placebo group (*p* = .02, Wilcoxon signed rank test). b: Principal Coordinates Analysis (PCoA) plot for the non-relapsed FMT-treated patients and placebo groups at week four (4 wk), showing a grouping of some FMT-treated patients with the donor. c: Percentage of OTUs that were present in the donor’s and patients’ post-FMT sample but absent in the patients’ pre-FMT sample, indicating the donor-like OTUs appearing after baseline in the FMT group. d: PCoA plot for the four FMT-treated patients who did not relapse during the one-year follow-up. The microbiota composition of two patients showed a shift toward the donor microbiota. Non-relapsed (nr) and relapsed (r) patients.

To further assess the engraftment of the donor’s microbiota, we analyzed the amount of luminal OTUs that were potentially donor derived. On average, 30.14% (±16.03) of the luminal OTUs that were only present in the donor samples and absent from the pre-FMT patients’ sample were found in the non-relapsed patients’ luminal microbiota at week four (Figure 3c). Although the average proportion of donor derived OTUs remained at almost the same level throughout the follow-up in the non-relapsed patients, there were individual differences between patients (Supplementary Figure S2, Additional File 3). In addition, the level of the donor-like OTUs in the relapsed FMT-treated patients and all patients in the placebo group was investigated (Figure 3c). However, no significant differences were found between the patient groups. In total, four FMT-treated patients remained in remission during the whole follow-up period. Longitudinal analysis of their microbiota by PCoA with Bray-Curtis dissimilarity revealed that the microbiota composition shifted toward the donor’s microbiota, especially in two out of four patients (Figure 3d).

**Figure 4.**
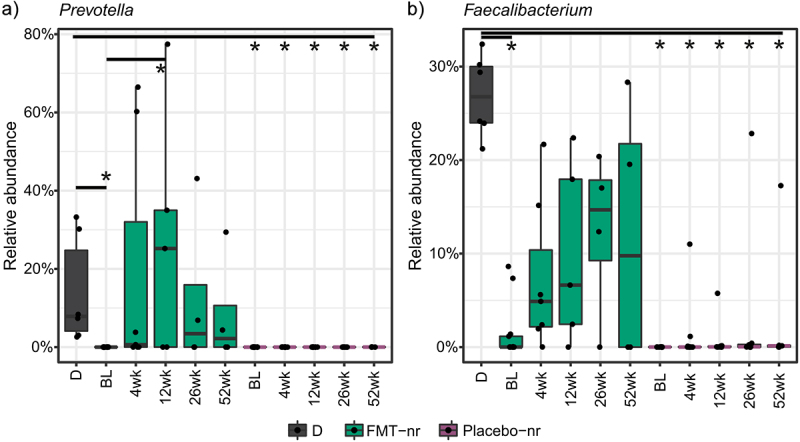
Post-FMT change in genus-level relative abundance.

Next, we aimed to identify bacterial taxa that would explain the higher microbiota similarity to the donor among the FMT-treated patients than patients in the placebo group. We found bacterial taxa that had a significantly different relative abundance between the donor and patients before FMT, but increased in abundance after FMT, and no significant difference was found in follow-up time points. Moreover, abundance remained low in the placebo group post-FMT (Supplementary table S5, Additional File 1). In particular, genera *Prevotella* and *Faecalibacterium* increased in abundance post-FMT, and seemed to persist in the patients’ microbiota for up to one year (Figure 4a,b). The abundance of *Prevotella* was also significantly lower at baseline compared to week 12 in the FMT-treated patients (Supplementary table S6, Additional File 1).

Selected genera *Prevotella* (a) and *Feacalibacterium* (b) had at baseline a significant difference in abundance between patients who would receive FMT and the donor, but no significant difference was found post-FMT. Patients in the placebo group had a significantly lower abundance of selected genera compared to the donor at all time points. Significant difference (q < 0.05) as compared to the donor is indicated with an asterisk. D = donor, nr = non-relapsed.

Overall, we detected a high variability in the patients’ microbiota during the follow-up period, in both the FMT and placebo groups. In the FMT group, microbiota modulation seemed to be more successful for some patients, but overall, similarity to the donor’s microbiota was modest.

### Mucosal microbiome composition had a high inter-individual variability

**Figure 5.**
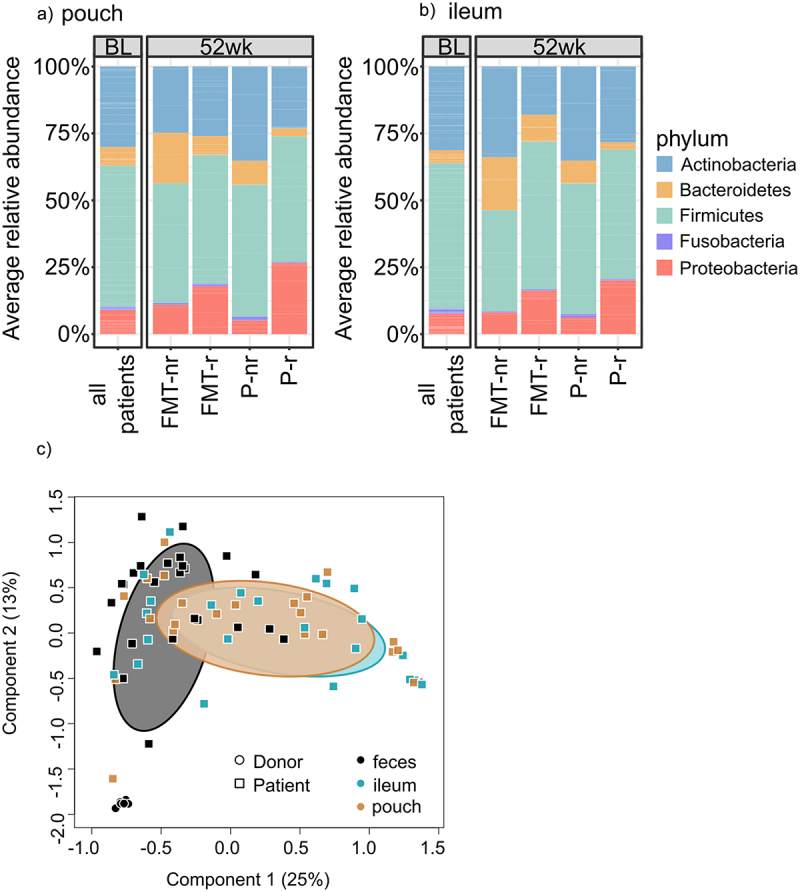
Average relative abundance of phylum-level taxa in the mucosal microbiota and difference between mucosal and luminal microbiota.

The patients’ mucosal microbiota in the pouch and ileum were surprisingly similar at baseline, and no significant differences in the average microbiota composition were detected between the two locations. At baseline, the composition in the pouch and ileum were dominated by phyla Firmicutes (52.76% ±21.95 and 52.45% ±23.03, respectively), Actinobacteria (30.10% ±23.69 and 31.27% ±23.63, respectively) and Proteobacteria (8.93% ±8.47 and 7.59% ±7.91, respectively) (Figure 5a,b). However, similar to the luminal microbiota, patients had a large inter-individual variation in their mucosal microbiota (Supplementary Figure S3&4, Additional File 3). Interestingly, the genus *Actinomyces* seemed to be enriched in the pouch and ileal mucosal in several patients at baseline and at 52 weeks, but the relative abundance of *Actinomyces* did not predict the clinical outcome (Supplementary Figure S5, Additional File 3). The overall microbiota did not differ at baseline in PCoA between patients who did not relapse and those who eventually relapsed; however, a few genus-level differences were identified. These included the genera *Robinsoniella*, *Natranaerovirga* and *Prevotella*, which had a higher relative abundance in the non-relapse patients, and the genus *Lautropia*, which had a lower level (Supplementary table S7A, Additional File 1). In the ileal mucosa, several genera belonging to the family *Lachnospiraceae* and genus *Clostridium IV* had a higher abundance. The family *Coriobacteriaceae* had a lower abundance in those patients who would not relapse than in those who would eventually relapse (Supplementary table 7 B, Additional File 1). Mucosal and luminal microbiota differed at baseline in PCoA between pouchitis patients (Figure 5c).

a: Phylum-level average relative abundance in pouch mucosa at baseline (BL) and at week 52 (52 wk). b: Phylum-level average relative abundance in ileal mucosa at baseline and at week. 52. nr = non-relapsed, *r* = relapsed. c: Principal Coordinates Analysis (PCoA) plot for the luminal and mucosal microbiota at genus-level with Bray-Curtis dissimilarity in the patients and the healthy donor showing separation of the mucosal and luminal microbiota in the patients at baseline.

After baseline, the next mucosal sampling took place one year later. First, we investigated whether the average mucosal microbiota in the pouch and ileum would differ at week 52. We found no statistical differences. Then, the mucosal microbiota in both locations were compared between FMT and placebo groups. In the pouch mucosa, the genus *Prevotella* had a higher relative abundance in the FMT group than in the placebo group (Supplementary table S7C, Additional File 1). Further, the genus *Natranaerovirga* had a lower relative abundance in the ileal mucosa in the FMT group than in the placebo group (Supplementary table S7D, Additional File 1).

To evaluate the possible FMT modulating effect on mucosal microbiota, we searched for differences in the mucosal microbiota between the non-relapsed FMT-treated patients and the placebo group. The number of non-relapsed patients was relatively low – four in the FMT group and five in the placebo group. This comparison excluded the relapsed patients who restarted antibiotic treatment between the baseline and 52-week sampling period to manage their pouchitis symptoms. The genera *Prevotella, Blautia* and *Pseudobutyrivibrio* had a higher abundance, while the family *Lachnospiraceae* and genus *Fusobacterium* had a lower abundance in the pouch mucosal microbiota in the non-relapsed FMT patients compared to the placebo group (Supplementary table S7E, Additional File 1). In the ileal mucosal microbiota, the genus *Roseburia* had a higher relative abundance, but *Lachnospiraceae*, *Veillonellaceae*, *Bacteroides*, *Ruminococcus2* had a lower abundance in the ileal mucosal microbiota of non-relapsed FMT patients as compared to the placebo group (Supplementary table S7F, Additional File 1).

These results indicate that a large inter-individual variation was found in the mucosal microbiota composition. However, the average mucosal microbiota was notably similar in the pouch and ileum. No major differences in the average mucosal microbiota composition were observed at the end of the study. However, a small number of bacterial genera had a significant difference in the relative abundance between the non-relapsed FMT patients and placebo group.

### Host mucosal transcriptional profiles reflect the transition from ileal to colonic gene expression and limited FMT effect

**Figure 6.**
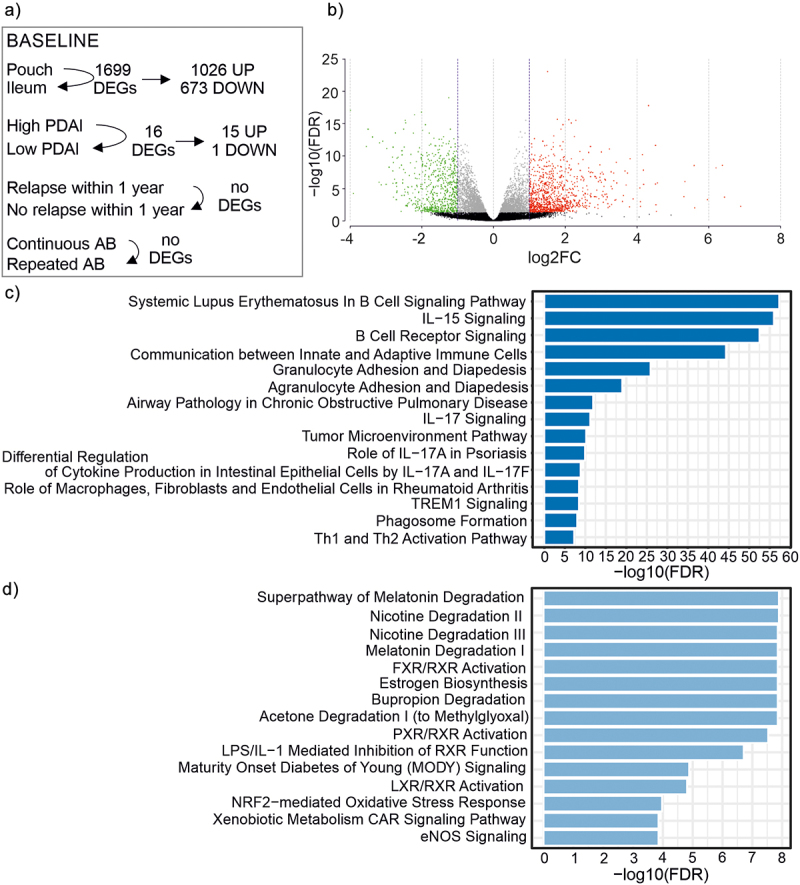
Baseline characteristics of mucosal gene expression.

RNA-sequencing was conducted to characterize the host mucosal gene expression profile at baseline, and to see how gene expression had changed during tissue regeneration after the pouch had been constructed from the terminal ileum. First, we identified the differentially expressed genes between the pouch (*n* = 26) and ileum (*n* = 26) at baseline. Altogether, we identified 1699 differentially expressed genes (q < 0.05) between the pouch and ileum (Figure 6a,b). These included 1026 upregulated genes (q <0.05, logFC >1.0) and 673 downregulated genes (q < 0.05, logFC <-1.0) in the pouch as compared to the ileum at baseline. The canonical pathway analysis was performed separately to the up- and downregulated genes, which revealed that several pathways related to inflammatory responses as well as both innate and adaptive immunity were significantly upregulated in the pouch mucosa as compared to the ileum (Figure 6c). The downregulated pathways included melatonin and nicotine degradation pathways, lipid and xenobiotic metabolism-related pathways, and a pathway related to oxidative stress response (Figure 6d).

a: Schematic representation of the differential gene expression analysis comparisons at baseline and the number of differentially expressed genes (DEGs). Comparison of pouch (*n* = 26) and ileum (*n* = 26), patients with high PDAI (≥7, *n* = 8) versus low PDAI values (<7, *n* = 18), patients who would eventually relapse (*n* = 17), independent of treatment group, and who would not relapse (*n* = 9), and patients who used continuous antibiotics (AB) before the trial (*n* = 12) and who used repeated antibiotics before the trial (*n* = 14). b: Volcano plot showing genes from the comparison of ileal and pouch mucosal gene expression with q-value >0.05 in black, q-value <0.05 in gray (q-value <0.05, log2FC < -1.0), in green and (q-value <0.05, log2FC >1.0) in red. c: The 15 most significant canonical pathways associated with upregulated genes in the pouch as compared to the ileum at baseline. d: The 15 most significant canonical pathways associated with downregulated genes in the pouch as compared to the ileum at baseline.

In addition, we aimed to determine whether our chronic pouchitis patients had a shift toward a more “colon-like” transcriptomic profile in their pouch as compared to the ileum. This was conducted by examining the previously reported 46 genes shown to serve as biomarkers for the loss of ileal function and gain of colonic function in the pouch.^14^ Nearly all (45/46) of these genes were differentially expressed between the pouch and ileum in our study, and all 46 showed a similar direction of change with previously reported results (Supplementary table S8, Additional File 1).

Next, we compared the baseline gene expression profiles in the pouch and ileum samples of those patients who would eventually relapse (*n* = 17) and those who would not (*n* = 9). This was done to investigate whether these patients had differing mucosal gene expression profiles at baseline that could be used to predict the clinical outcome. We did not detect evidence of gene expression profiles that would predict relapse (Figure 6a).

We evaluated how the transcriptome of the pouch mucosa differed between the patients with a more severe disease – measured with the pouchitis disease activity score (PDAI) – and those with less severe disease. While the level of PDAI value did not explain the variation in luminal or mucosal microbiota composition at baseline, a comparison between the patients with high PDAI (≥7, *n* = 8) and low PDAI values (<7, *n* = 18) at baseline resulted in 16 significant DEGs (q-value <0.05, Figure 6a). The 15 upregulated genes were: IL1RN, LUCAT1, DUSP4, CXCL8, INHBA, MMP10, KRT17, NMUR2, SH3PXD2A-AS1, KLK10, FAM25A, FOSL1, LAMC2, ANXA1, C1QTNF9 (Supplementary table S9, Additional File 1). Of these, IL1RN and CXCR8 are related to immune or inflammatory response, and DUSP4, FOSL1, and LAMC2 are related to, for example, cell proliferation and differentiation. One downregulated gene was PKD1L2, which encodes a polycystin protein family member (Supplementary table S9). On the other hand, although we observed differences in baseline luminal microbiota composition between patients who used continuous (*n* = 12) or repeated (*n* = 14) antibiotics before the study, we did not find DEGs between these same patients (Figure 6a).

Together the DEG results at baseline show that the pouch mucosa has undergone a transition from ileal- to colonic-type gene expression, and that disease activity was associated with the upregulation of immune and inflammatory response along with cell proliferation and differentiation.

We aimed to investigate the differential gene expression in the host mucosa samples collected at baseline (*n* = 26) in comparison with the ones collected at 52 weeks (*n* = 26) and between the intervention groups. However, no DEGs were found when comparing the pouch samples from baseline and 52-weeks within the FMT or placebo groups (*n* = 13, both groups). Moreover, the comparison of pouch samples (*n* = 26) between FMT and placebo groups at 52-weeks resulted in no DEGs.

One year after the intervention we aimed to investigate the differential gene expression in the host mucosa compared to the baseline and between the intervention groups. However, no DEGs were found in the pouch within the FMT or placebo groups. Similarly, no DEGs were found in the comparison between the FMT and placebo groups at 52-weeks in the pouch.

Next, to address the possible FMT-induced changes to the pouch mucosal gene expression at 52 weeks, we performed DEG analysis among the patients who did not relapse during the follow-up, and compared their gene expression at 52 weeks to that at baseline. We analyzed the DEGs between baseline and 52 weeks from the FMT (*n* = 4 non-relapsed) and placebo (*n* = 5 non-relapsed) groups separately. There were four significantly expressed genes (q < 0.05) in both separate comparisons. In the FMT group, PAX8-AS1 was upregulated, while CXCR4, C21orf62 and FGF19 were downregulated at 52 weeks as compared to the baseline. In the placebo group, TNFRSF11B and WNT5A were upregulated, while CXCR4 and AC012459.1 were downregulated at 52 weeks as compared to the baseline. Interestingly, CXCR4, which encodes a chemokine receptor 4, was downregulated at 52 weeks in both groups. When all non-relapsed patients (*n* = 9) were compared together, the same trend was observed for the gene CXCR4 as in the comparison for both study groups separately, although the difference was not significant.

One year after the intervention, only minor differences in gene expression were found. The gene CXCR4 was downregulated in the FMT and placebo group among the non-relapsed patients.

## Discussion

This study evaluated the microbiota changes in both the lumen and mucosa during a one-year follow-up of FMT-treated chronic pouchitis patients. The study provides novel long-term perspectives into pouch microbiota, as well as microbiota modulation findings from the first completed blinded and placebo-controlled clinical trial on FMT for pouchitis. To support the microbiota findings, host RNA expression was evaluated to investigate host mucosal gene expression profiles at baseline and 1 year after the intervention was provided.

In this study, patients’ baseline samples showed a high variation between subjects, a low diversity, and an altered microbial composition when compared with a healthy donor. Our results support those of several other previous studies, which profiled pouchitis microbiota and reported significant inter-individual variation in the patients’ microbiota.^41–43^ Furthermore, our results are in accordance with the previous findings of characteristic IBD and pouchitis microbiota, which underline key aspects such as reduced bacterial diversity and compositionally imbalanced microbiota when compared to healthy individuals.^12,44–46^ For example, a higher relative abundance of *E. coli* and other potentially pro-inflammatory bacteria and a lower relative abundance of *Faecalibacterium prausnitzii* have been observed in IBD patients, and especially in pouchitis patients, as compared to the healthy individuals.^47^

Differences between the healthy colon environment and ileal pouch, e.g., oxygen status, pH, transit time, as well as recurrent inflammation periods and common use of antibiotics in the pouchitis patients are likely to explain the observed differences. We found perturbations introduced by antibiotics in the patients’ baseline microbiota profiles in this study. Prior continuous use of antibiotics was linked to a shorter relapse-free period in the FMT-treated patients.^21^ Although the continuous use of antibiotics to manage symptoms may suggest a more severe disease due to higher host responsiveness to microbiota, the deeper microbiota dysbiosis at baseline also may have contributed to the microbiota modulating effect of FMT and the clinical outcomes.

Similar to the luminal microbiota, the mucosal microbiota composition had a high inter-individual variation. Further, the ileal and pouch microbiota compositions did not differ significantly. This was in line with the study by Morgan and colleagues^48^ who reported that microbiota variation between individuals was higher than the variation between the two locations. Interestingly, a proportion of patients had a high abundance of *Actinomyces* in their mucosal microbiota. Species in the genus *Actinomyces* are obligate anaerobes which are known to inhabit different parts of the human body, such as the oral cavity and intestinal tract, but can also be causative agents of actinomycosis, especially in immunocompromised patients.^49^ To our knowledge, there are no previous reports on the high amounts of *Actinomyces* in the mucosal microbiota among pouchitis patients. However, there was no association between the clinical outcome of patients and the abundance of *Actinomyces*. Hence, the possible role of *Actinomyces* in pouchitis remains obscure.

Overall, we could not confirm the association between the higher engraftment of the donor’s microbiota and better clinical outcome due to a small number of non-relapsed patients. In the current study, patients had a high variation in microbiota composition and unstable bacterial profiles during the one-year follow-up, in both the FMT and placebo groups. This instability is in line with the longitudinal study of UC patients’ microbiota, which reported low microbial stability even during remission.^50^ Despite the observed variation in the microbiota, the non-relapsed FMT-treated patients after the first FMT had significantly more similar overall microbiota composition with the donor than the placebo-treated patients. In particular, two patients showed a clear shift toward the donor’s microbiota, suggesting that there may be an individual response to the treatment. However, the poor engraftment of the donor’s microbiota in this study is in line with several earlier studies using FMT in pouchitis that have also reported a poor or moderate engraftment of the donor’s microbiota.^41–43,51^ On the contrary, a study with a multi-donor approach and an intensive 14-day treatment with daily FMT-enemas resulted in a relatively high level of donor engraftment.^52^ Nevertheless, no clear association with a better clinical outcome was reported.

It is clear that the niche differences between a healthy colon and a pouch can limit the engraftment, and that inflammation as such can create conditions that are unfavorable for certain microbes to colonize.^3,53^ Higher donor’s microbiota engraftment has been associated with better clinical outcomes in response to FMT for UC patient.^54,55^ However, it is not clear whether remission favors better colonization or colonization induces/maintains remission and thus causality still remains to be demonstrated. These findings suggest that engraftment of the donor microbiota to pouchitis patients is more complex than what has been observed in other indications, for example, rCDI and irritable bowel syndrome (IBS), where a high level of microbiota similarity to the FMT donor has been observed.^56–60^

The ‘super-donor’ effect has been observed in FMT studies, specifically in the treatment of UC and IBS.^54,61^ The term ‘super-donor’ has been used for donors whose recipients have significantly more successful clinical outcomes after FMT than recipients from other donors.^62^ Herfarth and colleagues^51^ attempted donor pre-selection for pouchitis by choosing a donor with a microbiota abundant in butyrate-producing bacteria, but the results were rather disappointing. The donor of our study was a healthy female who was carefully screened in terms of safety aspects for eligible fecal donors, but she was not pre-selected in the sense of compatibility for chronic pouchitis, as there are no such standards available.

We identified a few bacterial genera such as *Prevotella* and *Faecalibacterium* that were enriched in the luminal microbiota of the FMT-treated patients, and were potentially donor derived. It is noteworthy that one year after FMT, *Prevotella* levels were also increased in the mucosal microbiota of FMT-treated patients as compared to the placebo group, indicating that *Prevotella* engraftment may have been long term. In general, species from the genus *Prevotella* are common inhabitants in several human body sites including the intestinal tract. While genus *Prevotella* has been associated with high fiber diet,^63^ it had also been associated with inflammation, and for example, rheumatoid arthritis.^64,65^ In mice, Prevotella spp. has been shown to induce inflammation.^66^ but no association between *Prevotella* and IBD pathogenesis has been found in humans.^65^ Thus, it is unclear whether the increase of *Prevotella* should be considered as a favorable microbiota modulation effect by FMT in the context of pouchitis. On the other hand, the genus *Faecalibacterium* includes the intensively studied *F. prausnitzii*, which is known for its butyrate producing capability and anti-inflammatory properties. It has been found to be depleted in IBD and pouchitis, and thus its increase could be regarded as a beneficial microbiota modulation effect.^47,67–71^

Our analysis on the patients’ mucosal transcriptional profiles showed that the pouch mucosa had undergone transition from ileal- to colonic-type gene expression during tissue remodeling. These results are in line with the study by Huang and colleagues^14^ who showed that the pouch undergoes a transcriptomic reprogramming, where a more colonic expression profile arises in the ileal pouch early after IPAA surgery. However, we did not find evidence of a predictive gene expression profile at baseline that would have explained the patients’ clinical outcome following the FMT treatment.

The pathway analysis revealed that inflammation along with innate and adaptive immunity-related pathways were upregulated in the pouch as compared to the ileum at baseline. These results support the idea that activation of inflammatory pathways, also during the quiescent periods of the disease, may underlie a disturbed host-microbiota interaction in chronic pouchitis.^72^ The downregulation of lipid and xenobiotic metabolism-related pathways, as well as degradation of melatonin and nicotine-related pathways is in line with the study by Huang et al.^14^ It can be suggested that the mucosal transcriptomic profiles showed patterns similar to those previously reported for pouchitis patients. Moreover, in our cohort the patients with a higher PDAI value at baseline showed upregulation in inflammation and immunity, as well as cell proliferation and differentiation-related pathways. This finding indicates that further activation of host defense mechanisms along with tissue regeneration and repair occurs during relapse episodes.

We aimed to unravel the potential effect of FMT treatment on the host mucosal transcriptional profile, but after one year no differential gene expression was found between the non-relapsed FMT patients and the placebo group. However, we found that CXCR4 was downregulated in both study groups among the non-relapsed patients at 52 weeks as compared to the baseline. The gene CXCR4 encodes for a transmembrane G protein-coupled receptor for chemokines, and is widely expressed both in hematopoietic and non-hematopoietic tissues, such as the liver and gastrointestinal tract.^73,74^ This gene has several important roles during embryogenesis, and is essential in cell migration and immune responses. Interestingly, patients with inflammatory bowel disease showed an upregulation of CXCR4 and its ligand CXCL12; CXCR4 antagonists could reduce colonic inflammation.^75–77^ The observed decrease in the CXCR4 expression in the current study could be attributed to the prolonged period of remission regardless of which treatment group patients belonged to.

Here we presented the microbiota and host mucosal gene expression results from the first FMT-RCT study on pouchitis. The strengths of our study include the long (1-year) follow-up period with multiple luminal and mucosal microbiota samples. In addition, we were able to evaluate host mucosal gene expression from two time points. Limitations of the study include the relatively small cohort size (*n* = 26) and that additional microbial dietary supplements may add confounding factors to the analysis. Moreover, not all patients provided all luminal samples, or the samples were of low-quality, which limited the power of this study. Other researchers have also noted that it may be challenging to get high-quality data from pouchitis samples.^11^ Furthermore, our 16S rRNA gene amplicon analysis only reached genus-level taxonomic assignment, and strain-level differences among bacterial species can be significant to host–microbiota interactions.^63^ In addition, the potential functionality of microbiota, microbial metabolites, or members of the microbiota other than bacteria were not evaluated in this study.

To conclude, the two FMTs resulted in a relatively low engraftment of the donor’s microbiota in chronic pouchitis. Compositionally, the patients’ microbiota had a high inter-individual variation pre- and post-FMT, however, we found some evidence for the enrichment of potentially beneficial genera. Transcriptomics showed that pouch seemed to have converted from ileum to colon-like gene expression profiles, but transcriptional changes over the one year after FMT were few. Overall, FMT protocols, including patient and donor selection and matching should be better optimized for this group of patients to achieve better clinical outcomes.

## Supplementary Material

Additional_File2.docx

Additional_File3.docx

Additional_file1.xlsx

## Data Availability

The datasets generated and/or analyzed during the current study are available in the European Nucleotide Archive (ENA) repository with the accession number PRJEB52304 or by request.
