# Supplementary material for: Microbiota and mucosal gene expression of fecal microbiota transplantation or placebo treated patients with chronic pouchitis
Source: Gut Microbes. 2024 Jan 12;16(1):2295445. doi: 10.1080/19490976.2023.2295445 (PMC10793679; doi:10.1080/19490976.2023.2295445)
Supplement: Additional_File2.docx [file KGMI_A_2295445_SM1982.docx]

Additional file 2.

Supplementary Methods

Library preparation

Library preparation was conducted as follows: 22 ng of DNA was used for the first PCR in a 20 µl reaction. Phusion High-Fidelity PCR Master Mix with HF Buffer (Thermo Fisher Scientific, CAT#F531L) was used for both PCRs. First, 16S forward (S-D-Bact-0341-b-S-17) and reverse (S-D-Bact-0785-a-A-21) primers targeting the hypervariable region V3-V4 were used.1 The thermocycler program was 98°C for 1 minute, followed by 45 cycles of 98°C for 10 seconds, 64°C for 40 seconds and 72°C for 40 seconds, and concluded with a final extension step at 72°C for 10 min. The second PCR used the index primers, with 4.4 µl of the PCR products from the first PCR as templates. The program was the same as in the first PCR, but with 25 cycles. After the second PCR, the quality and size of the PCR products were confirmed by gel electrophoresis. The PCR products were then purified with bead purification (AGENCOURT® AMPURE® XP beads, Beckman Coulter Life Sciences, CAT#A63881), and the concentration of the purified amplicons were measured with Quant-iT™ dsDNA Assay Kits, high sensitivity (HS) (Invitrogen by Thermo Fisher Scientific, CAT#Q33120).

Bacterial DNA amplification

Bacterial DNA amplification was done by whole length 16S rRNA gene PCR (program: 98°C for 2 minutes; 20 cycles of 98°C for 20 seconds, 55°C for 20 seconds and 72°C for 40 seconds; and final extension at 72°C for 5 minutes) with forward primer (Amp-F) and reverse primer (Amp-R).2

Detailed methods for microbiota analysis

Functions in R package mare^3^ were used as follows: ‘ProcessReads’ function with forward reads trimmed from beginning at 20 bp, into read length of 180 bp, truncated, trunc. quality 2, filtered, max.expected.error = 1, min.read.abundance = 0.001%. ‘PCoA’ function was used for selected groups of samples with genus level relative data and ‘bray’ distance. ‘GroupTest’ function was used for selected groups of samples with relative data, outlier cutoff 3, p. cutoff 0.05, nonzero T.

1. Klindworth A, Pruesse E, Schweer T, Peplies J, Quast C, Horn M, et al. Evaluation of general 16S ribosomal RNA gene PCR primers for classical and next-generation sequencing-based diversity studies. Nucleic Acids Research. 2013 Jan 1;41(1):e1.
2. Wang RF, Beggs, Marjorie L., Robertson, Latriana H., Cerniglia, Carl E. Design and evaluation of oligonucleotide-microarray method for the detection of human intestinal bacteria in fecal samples. FEMS Microbiology Letters. 2002;213(2):175–82.
3. Korpela K. (2016). mare: Microbiota Analysis in R Easily. R package version 1.0. https://github.com/katrikorpela/mare; doi:10.5281/zenodo.50310.
