# Supplementary material for: Microbiota and mucosal gene expression of fecal microbiota transplantation or placebo treated patients with chronic pouchitis
Source: Gut Microbes. 2024 Jan 12;16(1):2295445. doi: 10.1080/19490976.2023.2295445 (PMC10793679; doi:10.1080/19490976.2023.2295445)
Supplement: Additional_File3.docx [file KGMI_A_2295445_SM1981.docx]

Additional File 3. Supplementary Figures


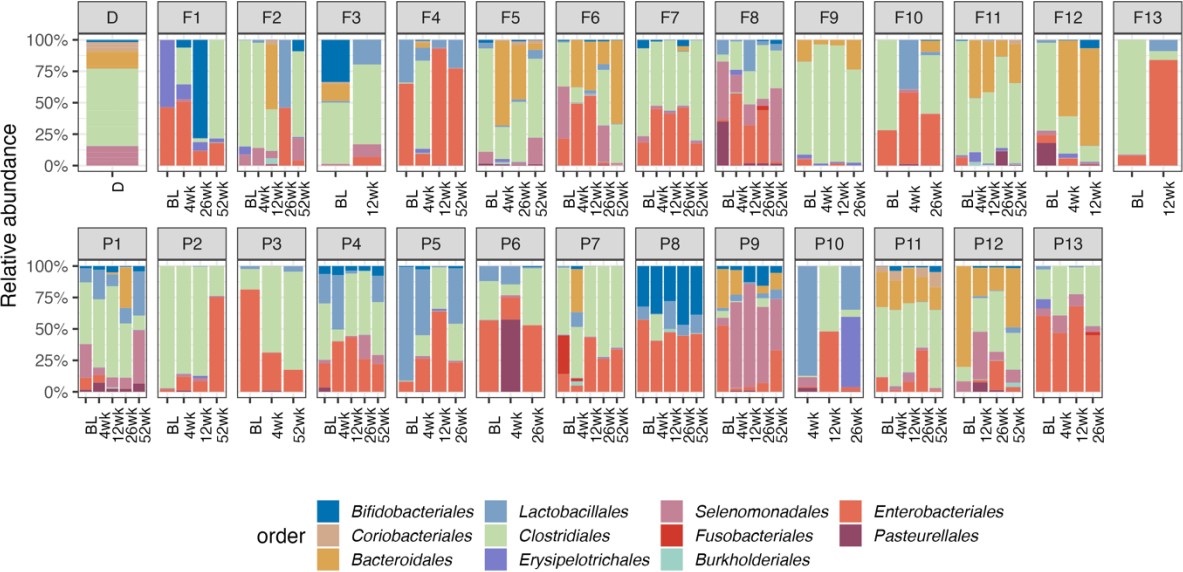


Supplementary Figure 1. Luminal microbiota changes during one-year follow-up in the FMT and placebo groups. Order-level average relative abundance in the donor (D) samples and patients (F1-F13) in the FMT group and in placebo group (P1-P13) at baseline (BL) and 4, 12, 26 and 52 weeks after FMT (4wk, 12wk, 26 wk and 52 wk).


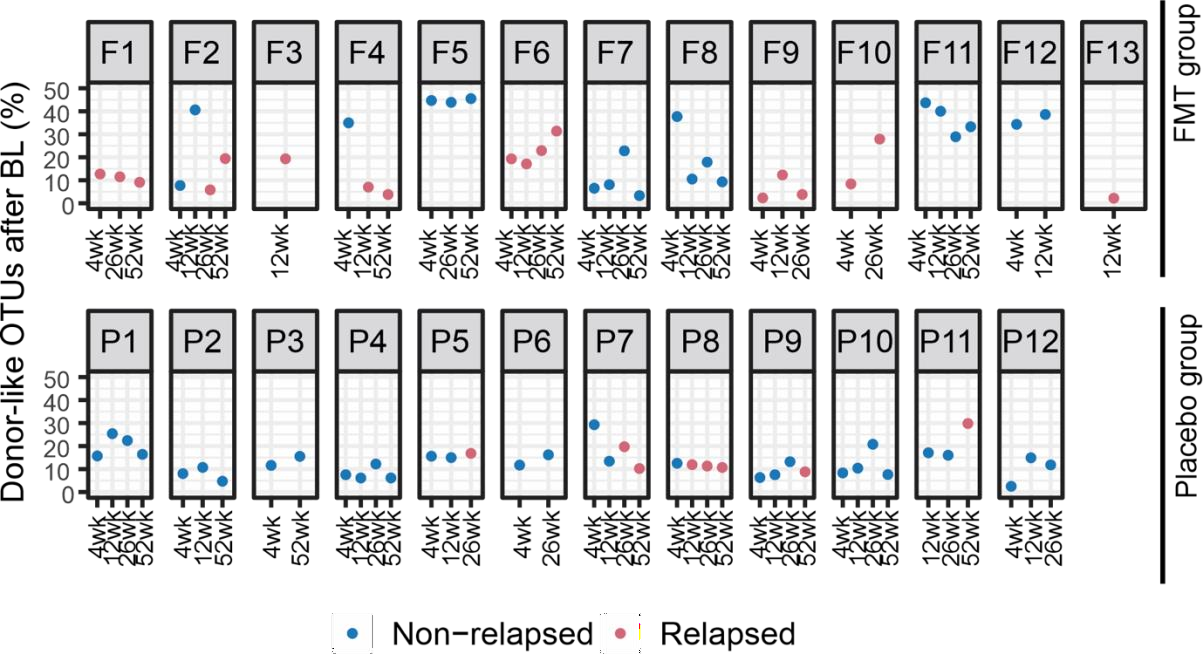


Supplementary Figure 2. Level of donor microbiota engraftment. Percent of OTUs that were present in the donor and patients’ post-FMT sample, but absent in patients’ pre-FMT sample. Patients in the FMT-group (F1-F13, top row) and in the placebo group (P1-P13, bottom row) and a clinical status marked with colors. Dark blue = non-relapsed, red = relapsed.


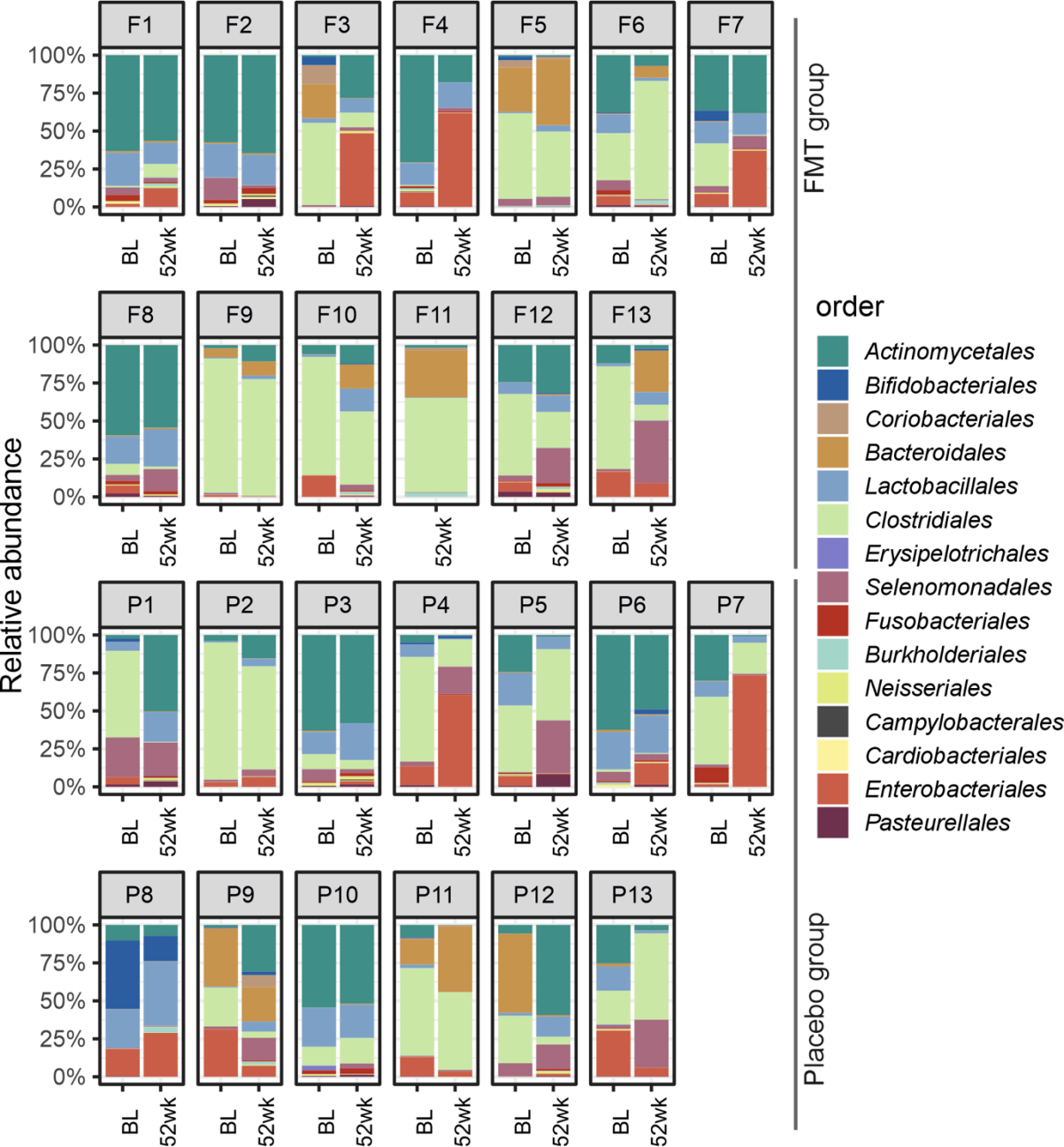


Supplementary Figure 3. Pouch mucosal microbiota composition. Order-level average relative abundance in the pouch mucosa at baseline (BL) and after 52 weeks (52wk) of the patients (P1-P26).


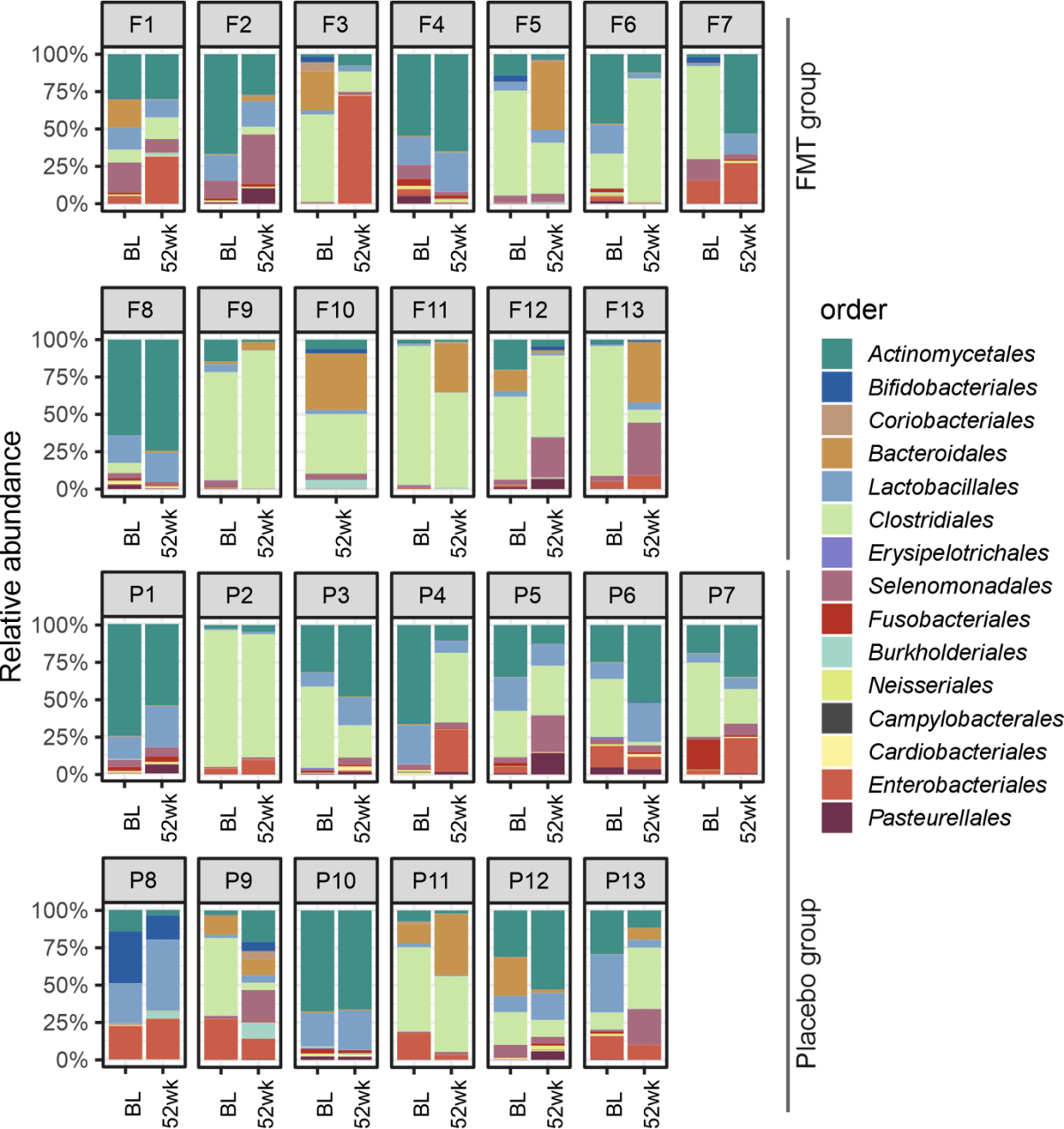


Supplementary Figure 4. Ileum mucosal microbiota composition of patients (P1-P26). Order- level average relative abundance in the ileum mucosa at baseline (BL) and after 52 weeks (52wk).


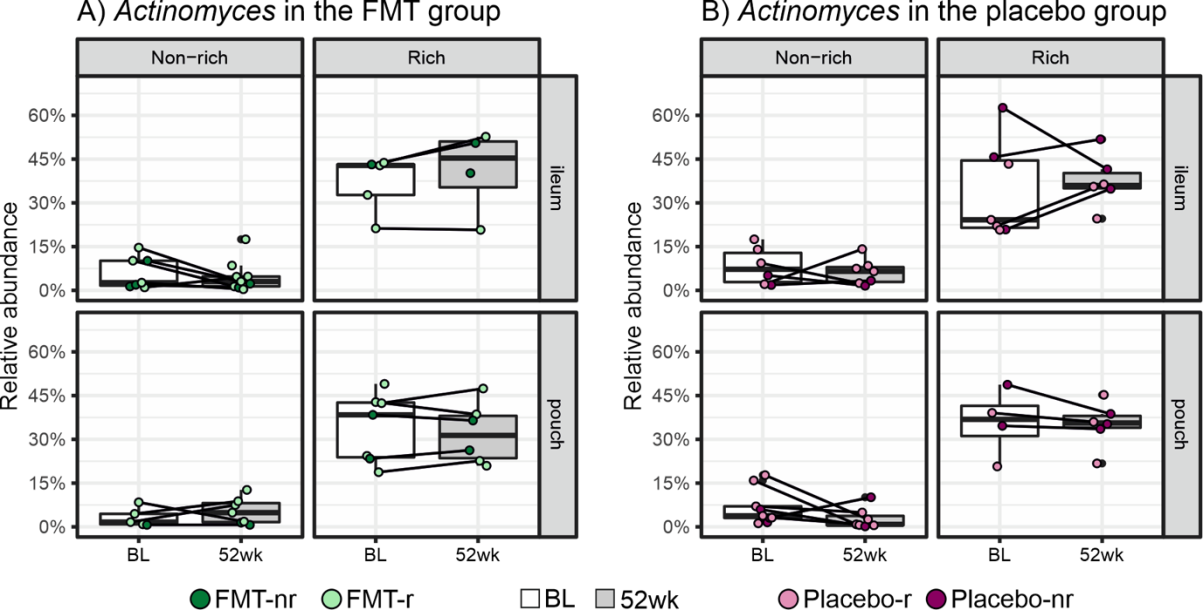


Supplementary Figure 5. Relative abundance of the genus *Actinomyces* in the mucosal microbiota. A) Abundance in the FMT group. B) Abundance in the placebo group. Paired samples from the same patient at two different time points are connected. Non-rich = relative abundance less than mean at baseline. Paired samples indicating a same patient are connected. Rich = relative abundance more than mean at baseline. Nr = non-relapsed, r = relapsed.
